# Supplementary material for: Epithelial-Mesenchymal Wnt Crosstalk Directs Planar Cell Polarity in the Developing Cochlea
Source: bioRxiv. 2026 Mar 27:2026.02.24.705389. Preprint. [Version 2] doi: 10.64898/2026.02.24.705389 (PMC13041925; doi:10.64898/2026.02.24.705389)

Supplementary Figure 1

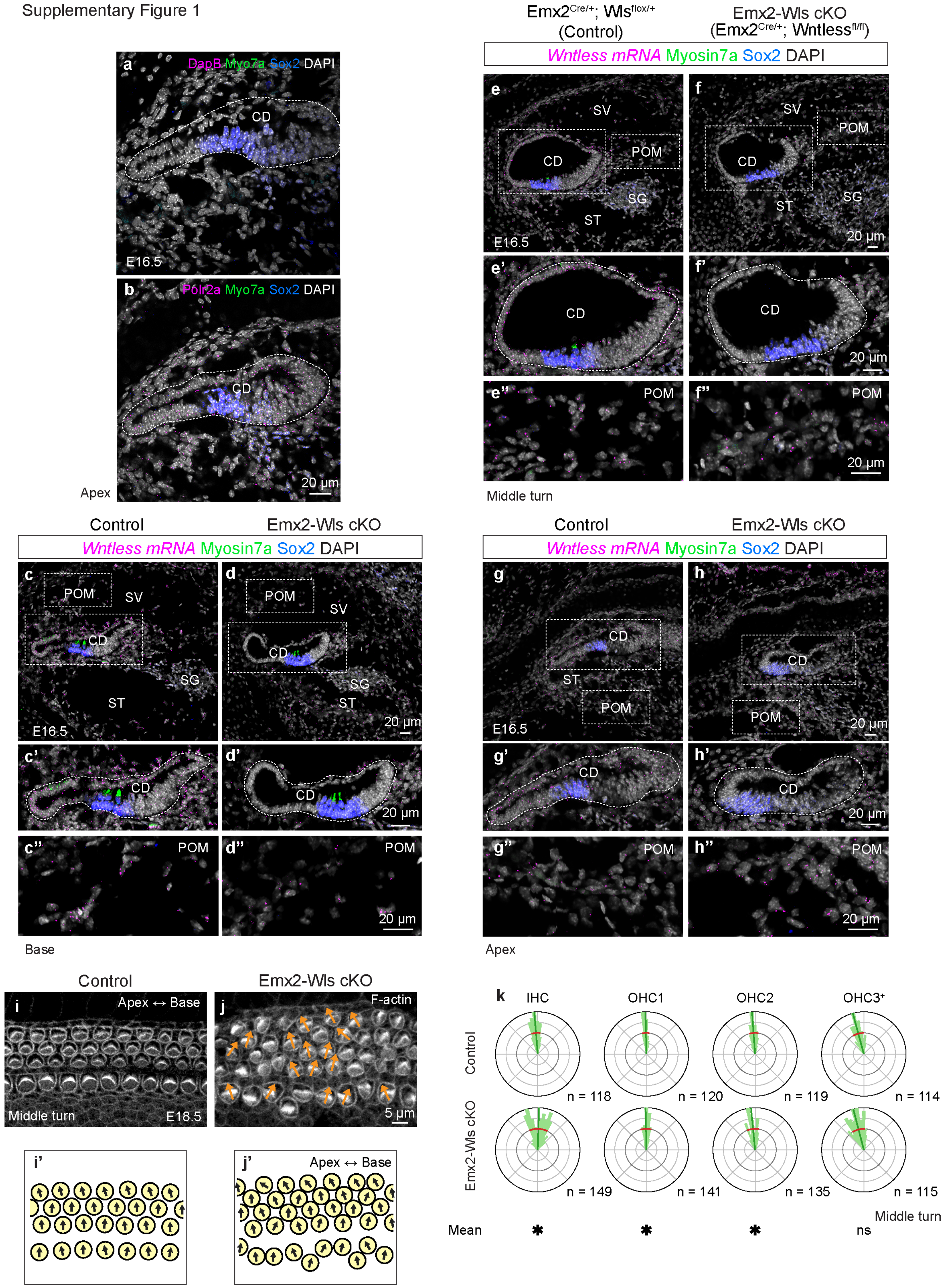

Supplementary Figure 2

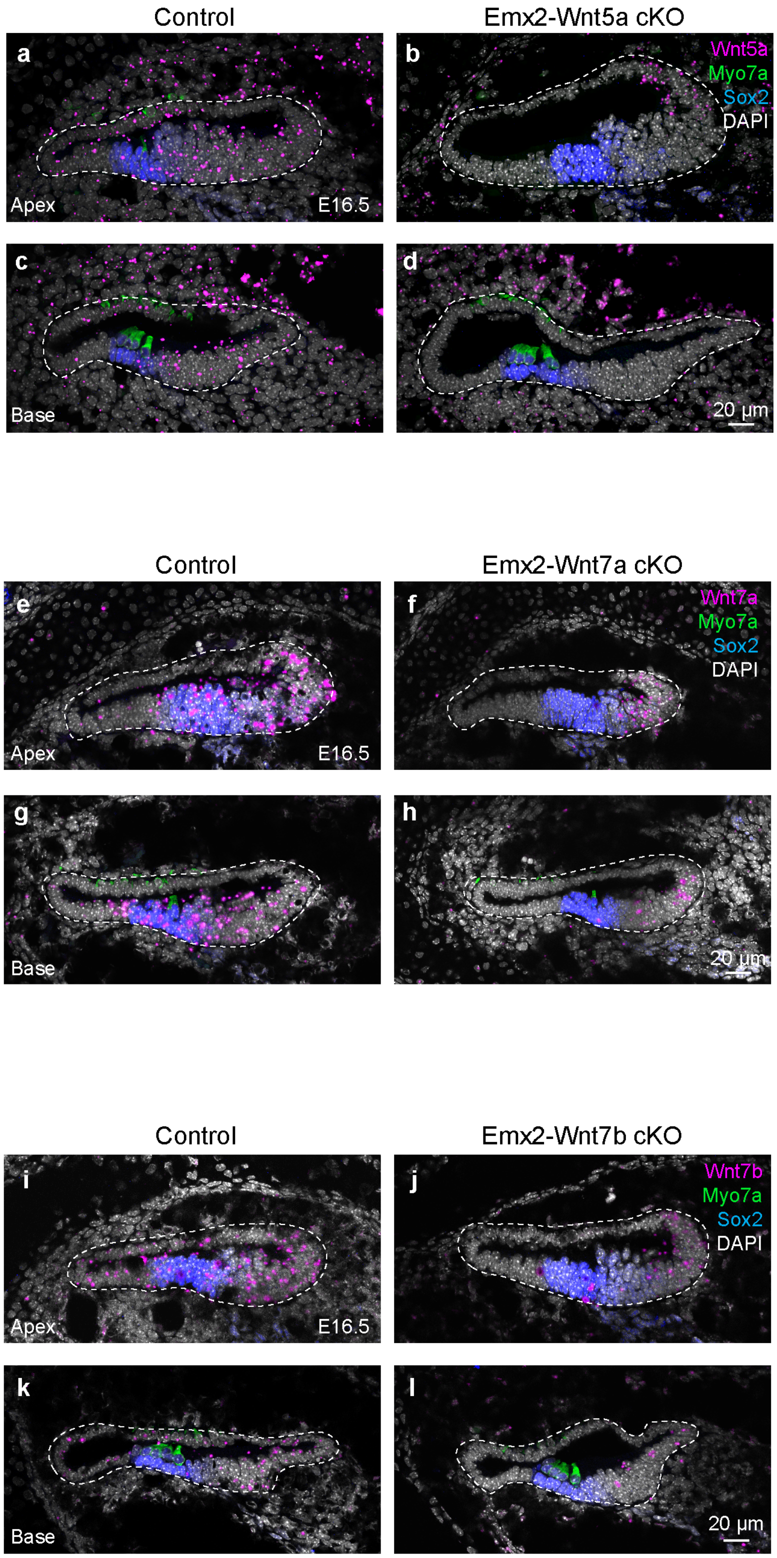

Supplementary Figure 3

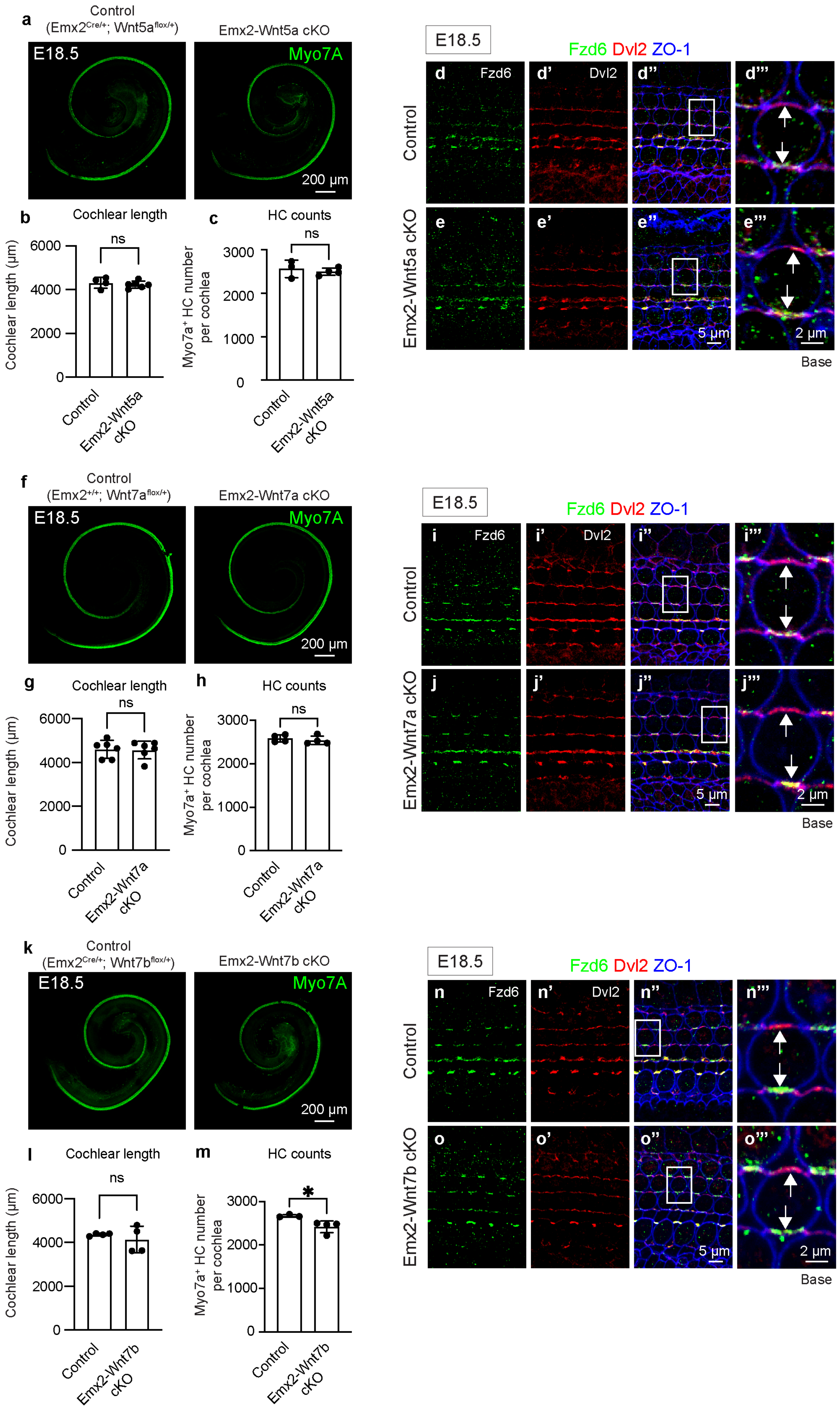

Supplementary Figure 4

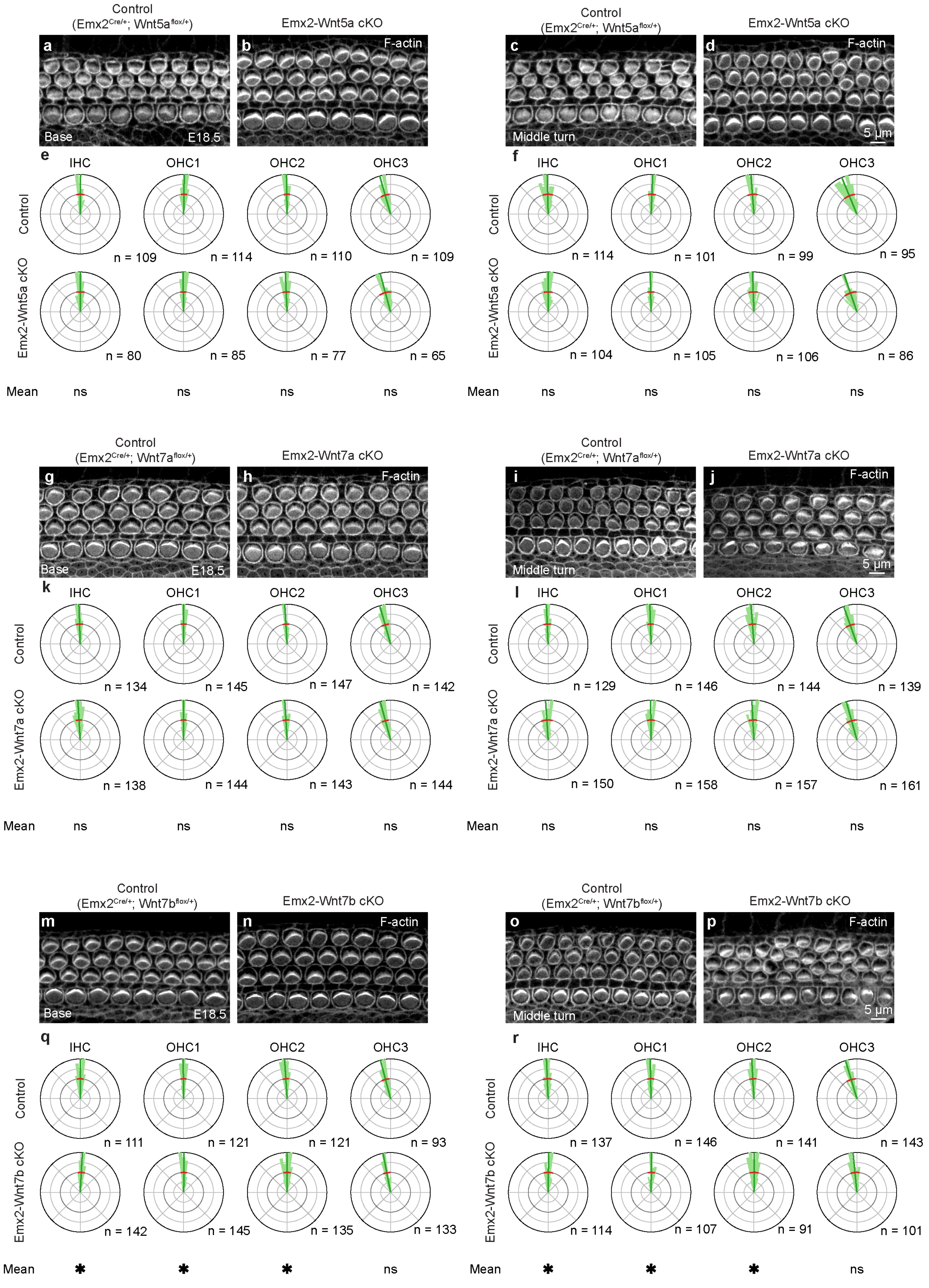

Supplementary Figure 5

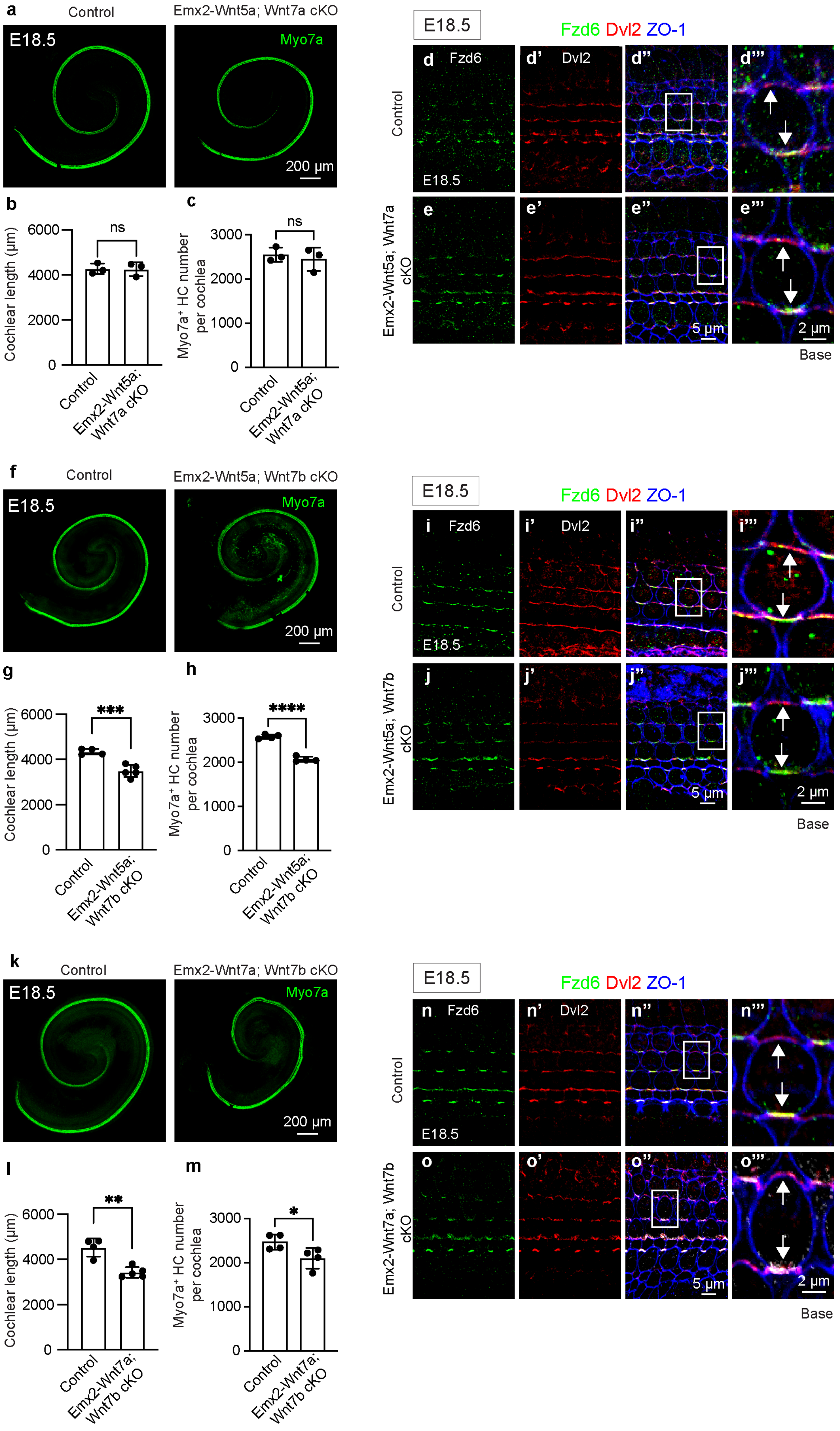

Supplementary Figure 6

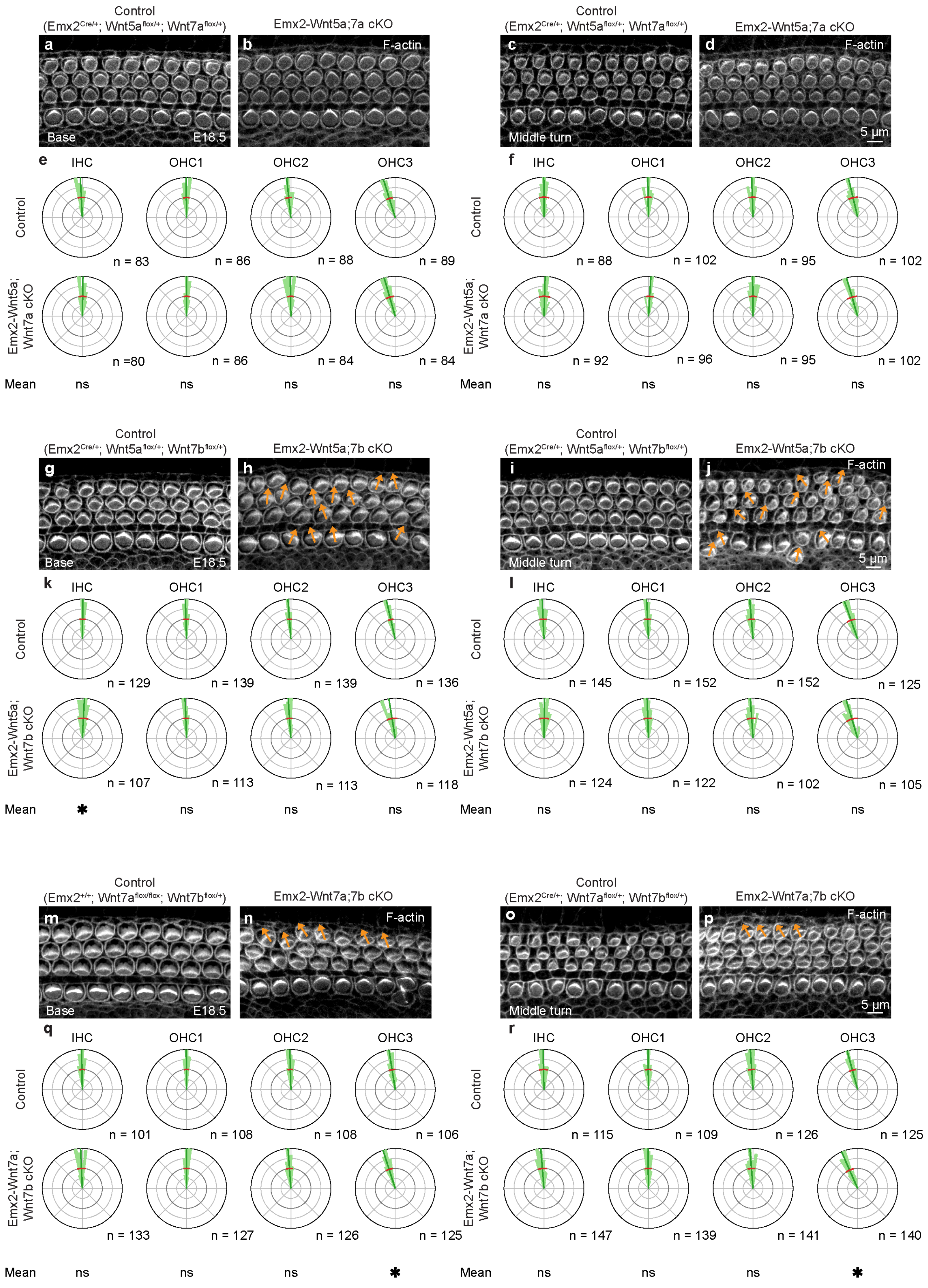

Supplementary Figure 7

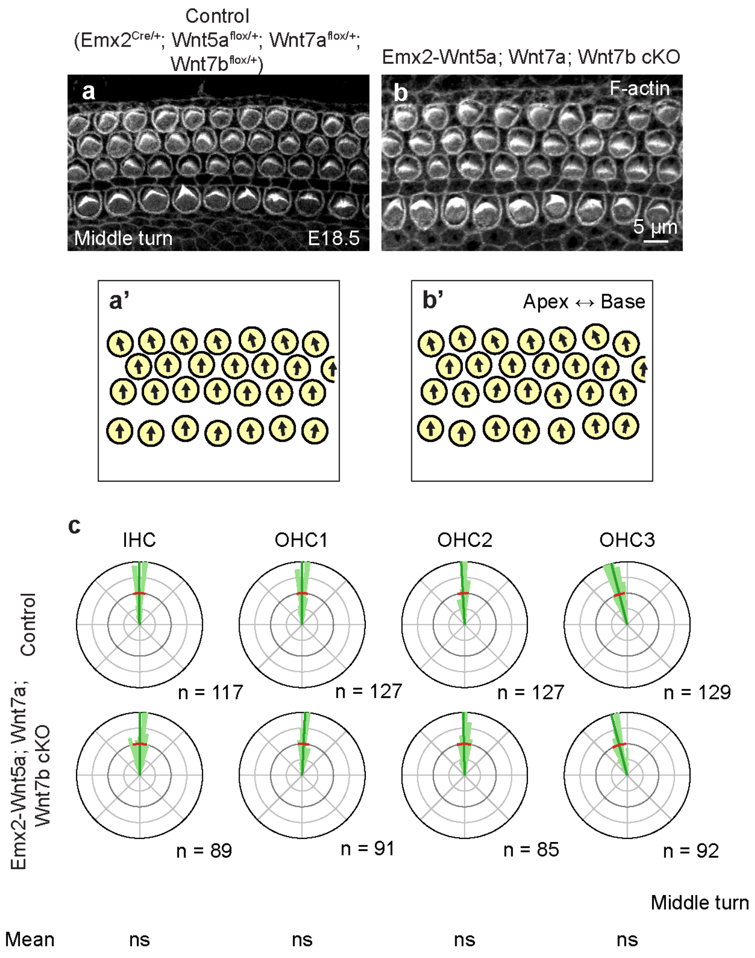

Supplementary Figure 8

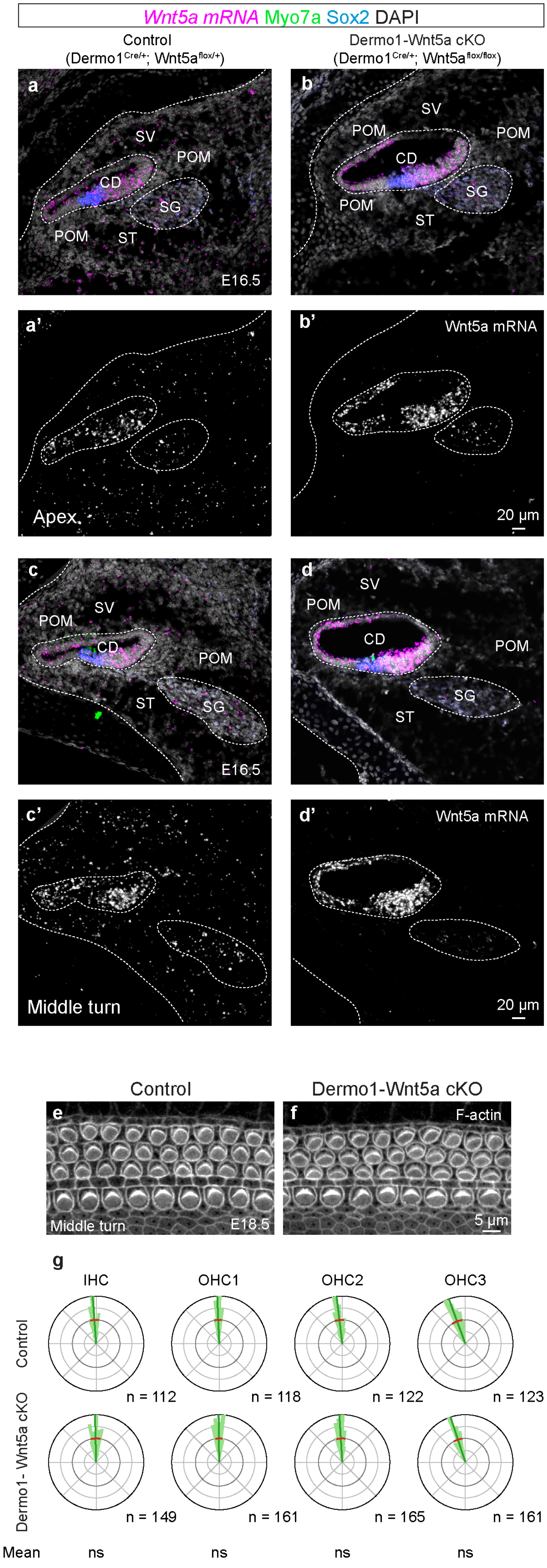

Supplementary Figure 9

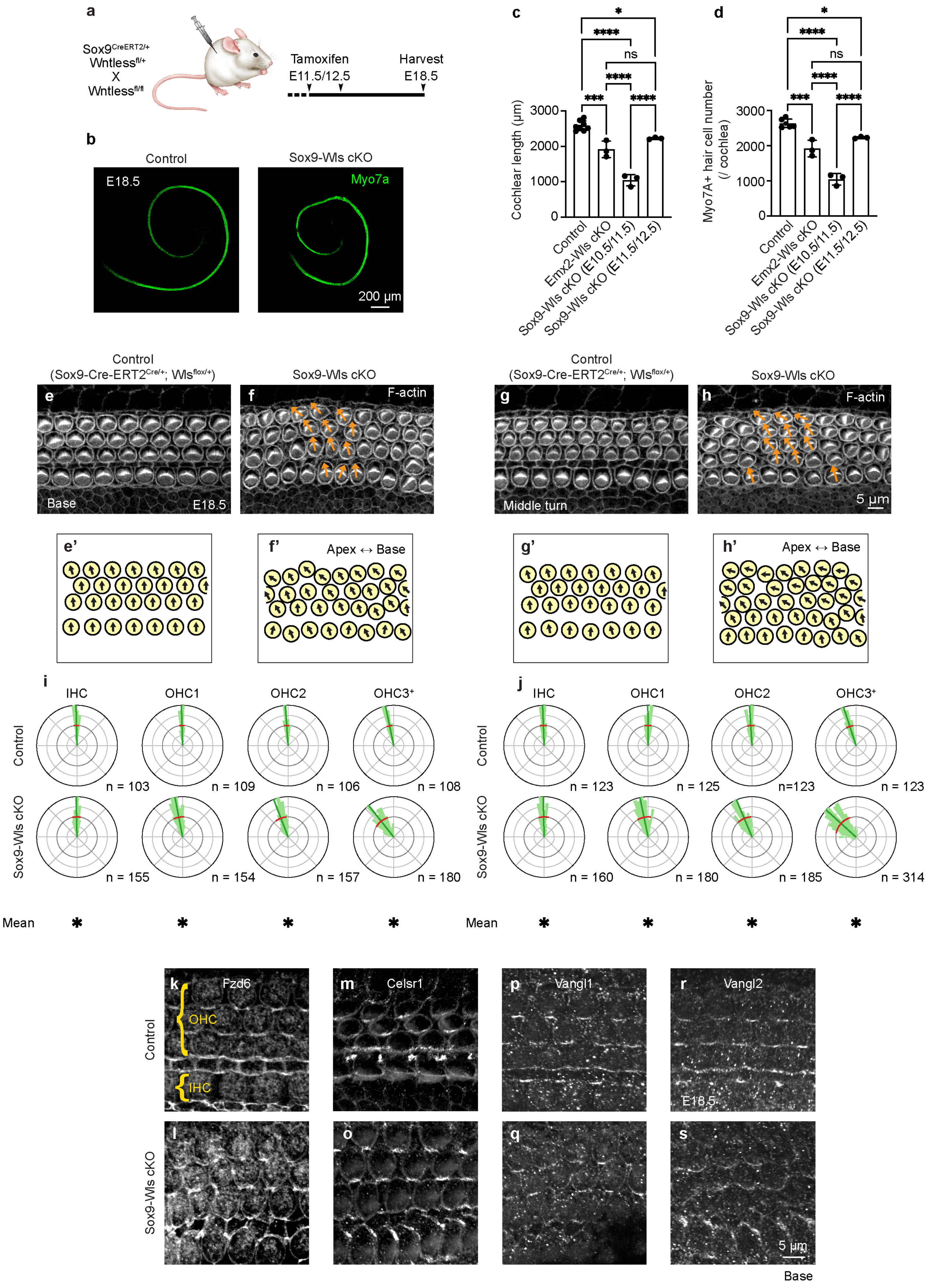

Supplementary Figure 10

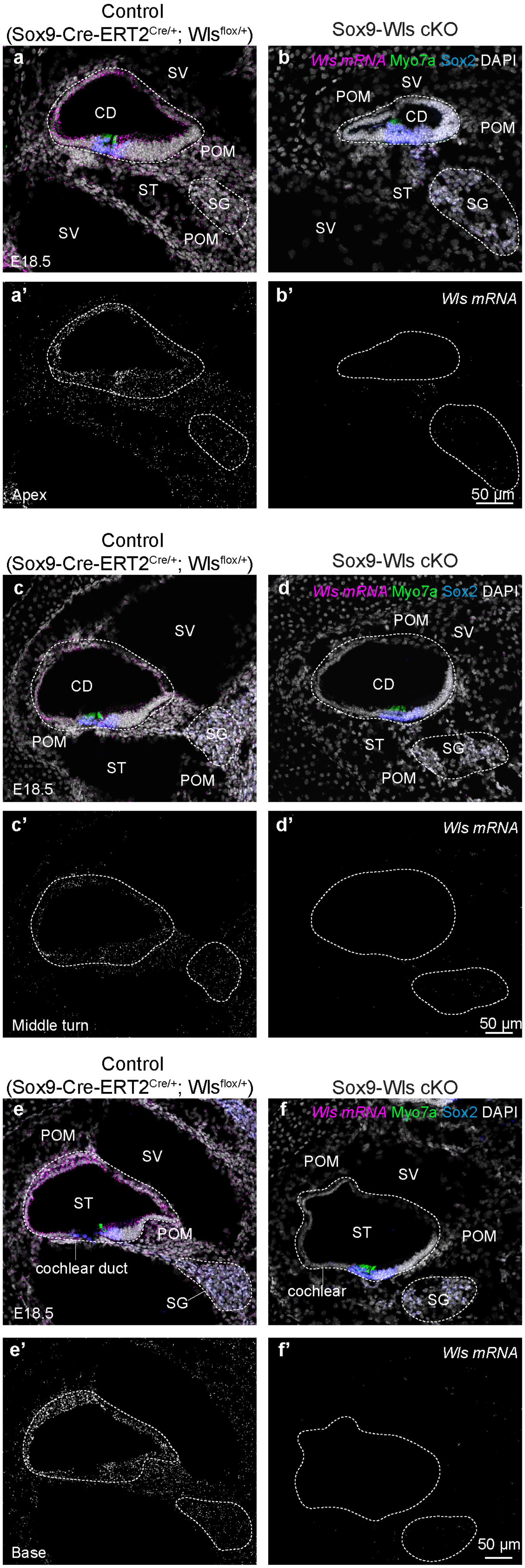

Supplementary Figure 11

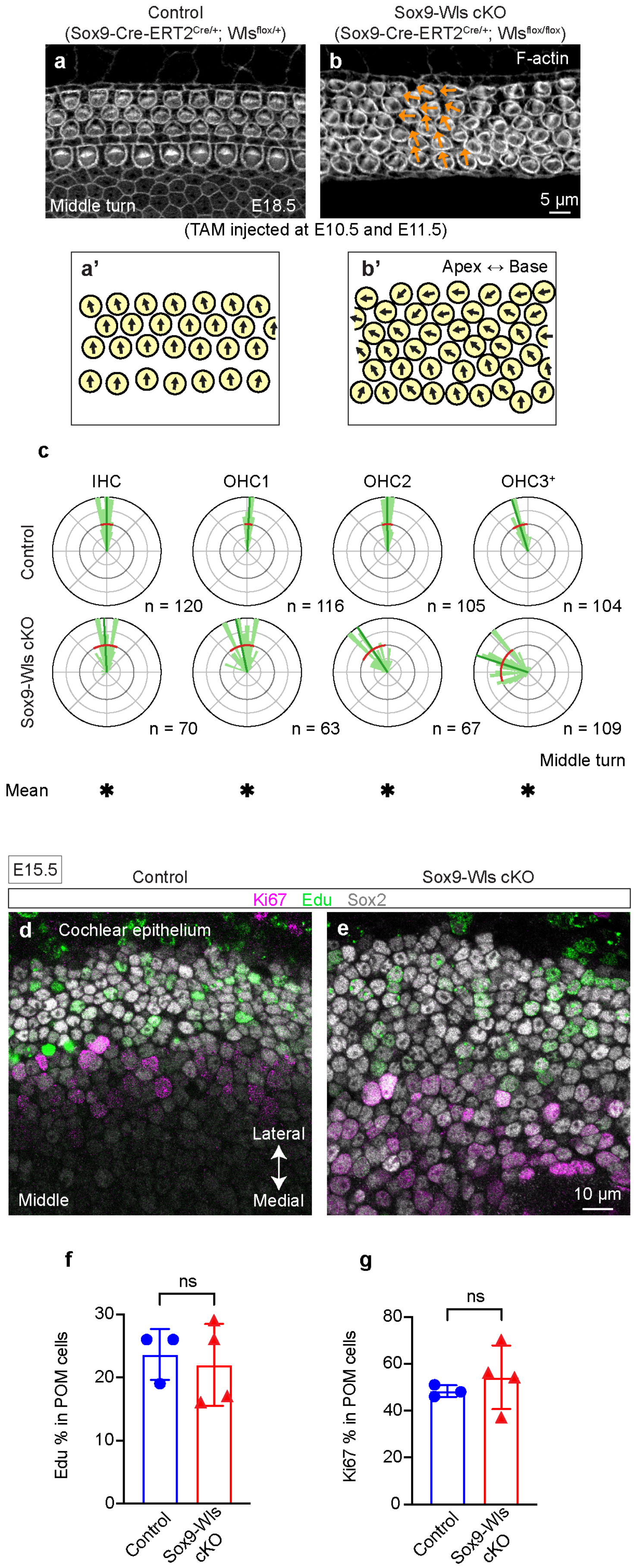

Supplement: Supplement 2 — Supplementary Figure 1. In situ hybridization and hair bundle orientation in the Emx2-Wls cKO cochlea. a-b. Negative (DapB) and positive (Polr2a) controls for in situ hybridization in E16.5 cochleae. c-h. RNA scope in situ hybridization of Wls mRNA in the basal, middle, and apical turn of the control (c-c’’, e-e’’, g-g’’) and Emx2-Wls cKO (d-d’’, f-f’’, h-h’’) cochleae. Magnified views (white boxes) of the cochlear epithelium and POM are shown in (c’-h’) and (c’’-h’’), respectively. In Emx2-Wls cKO cochleae, Wls mRNA is almost undetectable in the cochlear epithelium, while that in the POM remain comparable to those in control cochleae. i-j. Whole mount preparation of the Emx2-Wls cKO and control cochlea stained for f-actin. In the Emx2-Wls cKO cochlea, hair cells exhibit more variable orientations compared to controls. k. Circular histograms depicting the distribution of hair bundle orientation both control and Emx2-Wls cKO cochleae, showing significantly large variance and the average rotation for the IHC, OHC1, and OHC3 rows in the Emx2-Wls cKO cochleae. CD, cochlear duct; ST, scala tympani; SV, scala vestibule; SG, spiral ganglion. Scale bar:20μm in (a-h’’), 5μm in (I, j). n, number of hair cells measured. Circular mean and variance of hair cell orientation are indicated with blue and red lines in circular histograms, respectively. A Bayesian mixed model analysis used for comparison of circular mean of hair cell orientation between control and cKO groups. *significantly different (for hair cell orientation analysis), ns = not significant. Supplementary Figure 2. Expression of Wnt5a, Wnt7a, and Wnt7b mRNA in the control and conditional knockout cochlea. a-d. Wnt5a mRNA is robustly expressed in the epithelium and periotic mesenchyme of control cochlea (a, c) and is almost undetectable in the epithelium of Emx2-Wnt5a cKO (b, d) cochleae. e-h. Wnt7a mRNA is robustly expressed in the epithelium of the control cochlea (e, g) and is barely detectable in the Emx2-Wnt7a c [file media-2.pdf]
